# Supplementary material for: Nuclear Quantum Effects in the Ionic Dissociation Dynamics of HCl on the Water Ice Cluster
Source: Molecules. 2025 Jan 21;30(3):442. doi: 10.3390/molecules30030442 (PMC11820938; doi:10.3390/molecules30030442)
Supplement: Supplementary file 1 [file molecules-30-00442-s001.zip › molecules-3392897-supplementary.pdf]

Nuclear Quantum Effects in the Ionic Dissociation Dynamics of HCl on the Water Ice Cluster

Tatsuhiro Murakami *, Hinami Ueno, Yuya Kikuma and Toshiyuki Takayanagi *

Department of Chemistry, Saitama University, Shimo-Okubo 255, Sakura-ku, Saitama City 338-8570, Saitama, Japan; h.ueno.687@ms.saitama-u.ac.jp (H.U.); y.kikuma.323@ms.saitama-u.ac.jp (Y.K.)

* Correspondence: murakamit@mail.saitama-u.ac.jp (T.M.); tako@mail.saitama-u.ac.jp (T.T.); Tel.: +81-48-858-9113 (T.M. & T.T.)

Supplementary Material

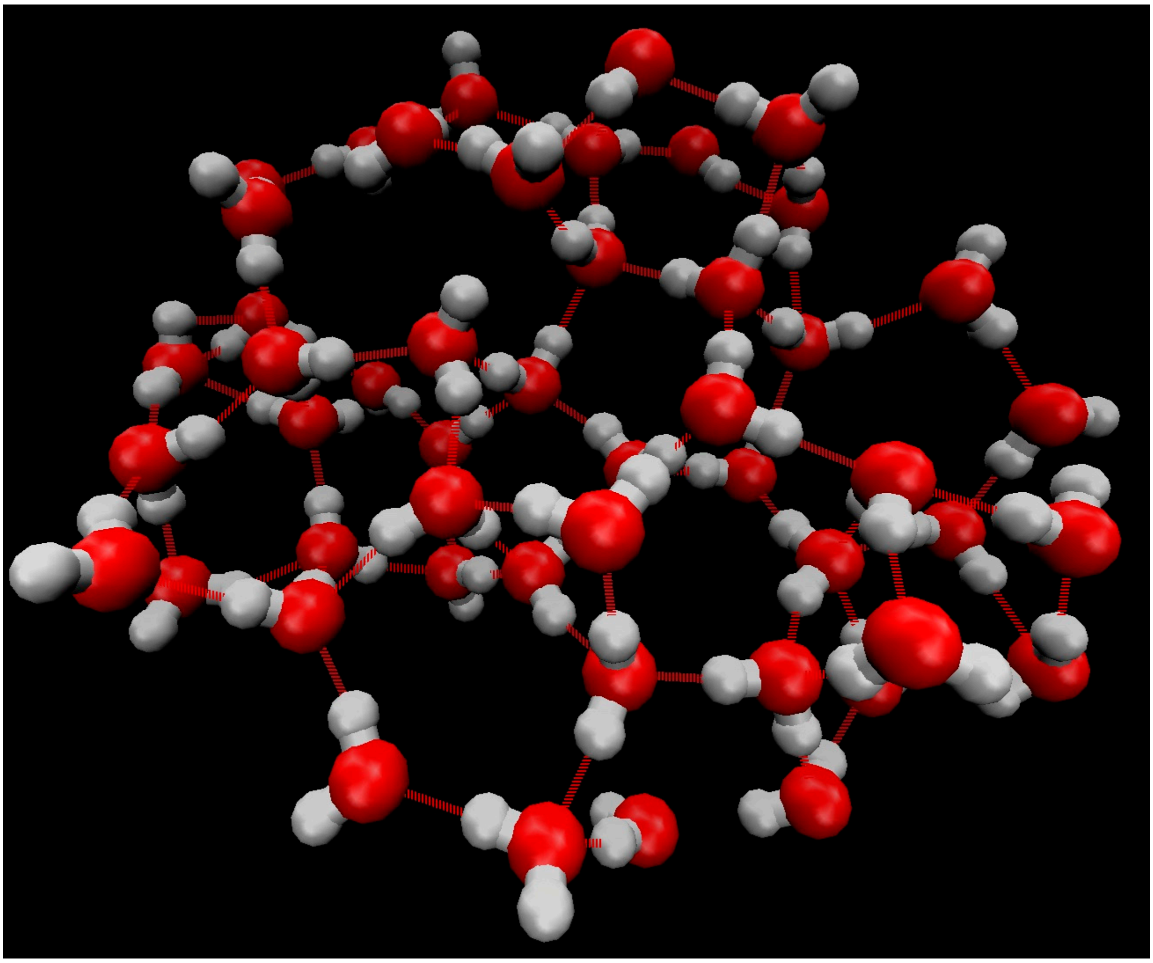

Figure S1. Initial amorphous water cluster structure

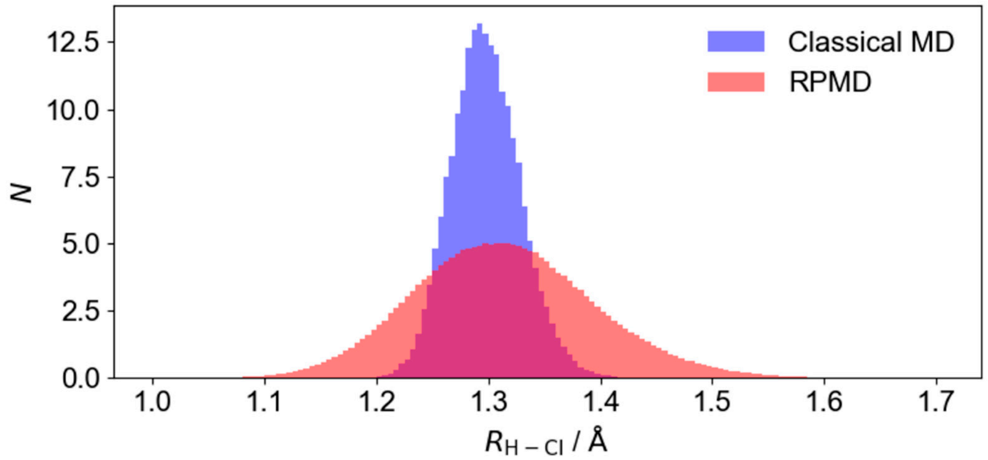

Figure S2. Probability densities of the isolated HCl molecule at 250 K using path-integral scheme with one bead (blue bars) and 16 beads (red bars)

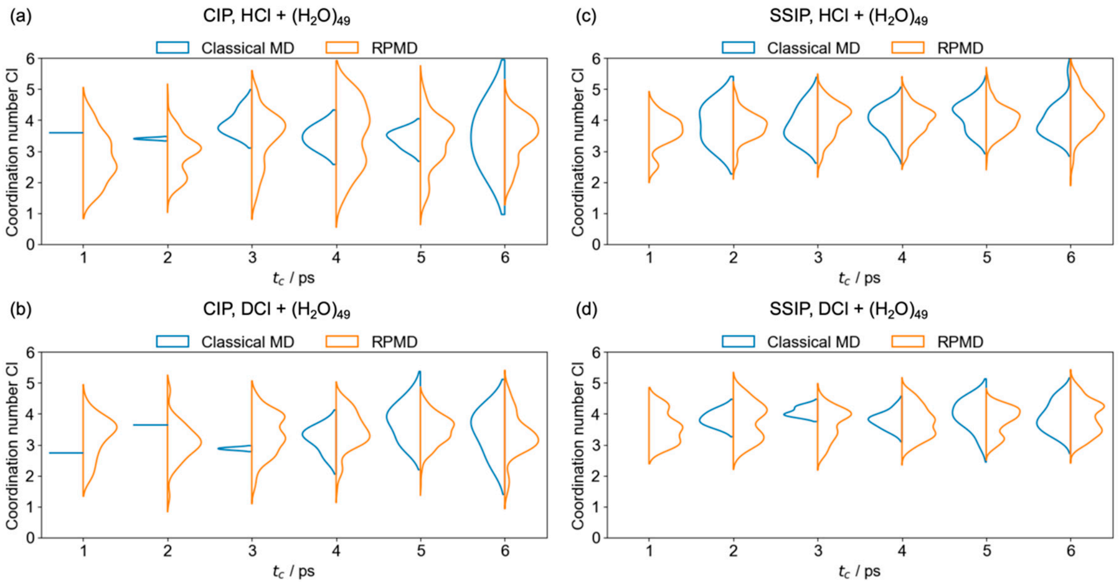

Figure S3. Time-evolved violin plots in terms of the coordination number of Cl in classical MD (blue) and RPMD (orange) for (a) HCl + $(H_2O)_{49}$ and (b) DCl + $(H_2O)_{49}$ in the CIP state, and for (c) HCl + $(H_2O)_{49}$ and (d) DCl + $(H_2O)_{49}$ in the SSIP state

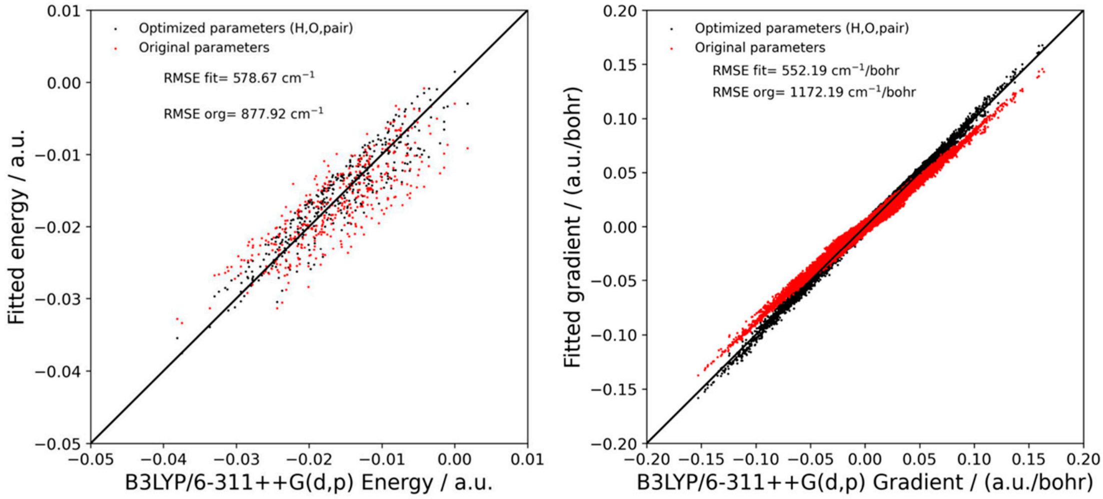

Figure S4. Root-mean-square errors (RMSEs) for the energy (left panel) and the gradient (right panel) calculated using the original (red dots) and optimized (black dots) GFN2-xTB method.

Table. S1. Cartesian coordinates (xyz format in Angstrom) of the water ice cluster shown in Figure S1

147

ANGSTROM

H	1.52960324	-4.53639507	5.39040852
H	1.23272014	-3.71324444	4.14877176
H	-0.81133992	-2.37405443	3.21439672
H	0.63025570	-1.89966071	2.70534921
H	-2.68076491	-1.45844460	2.20260072
H	-3.30852437	-1.74455965	3.57634759
H	-4.03537893	7.03427696	2.05462813
H	-3.30091619	6.03620481	1.11558795
H	-2.67696166	-3.75063109	2.25266027
H	-1.57380211	-4.70126486	1.78076267
H	1.93028402	-7.61704588	3.18420053
H	2.37885427	-6.22875261	3.68150115
H	0.14547934	-7.75329876	0.50740778
H	1.64509821	-7.82826996	0.89457321
H	0.42219096	-4.67343903	2.51358080
H	0.34505186	-6.18789625	1.96871936
H	-4.19409990	-7.10131884	0.60322541
H	-3.41024947	-6.08285952	1.48229551
H	-6.94277048	1.44604468	1.57273364
H	-6.98983192	1.27623999	0.03561893
H	0.71824026	4.17973804	1.51789093
H	-0.18377368	2.91134596	1.68242860
H	-3.31150913	-0.02095767	0.17379636
H	-1.89385152	0.22188287	0.69779015
H	1.10206985	-1.41508639	0.41245684
H	2.02305794	-0.41691196	1.11370420
H	0.21986422	0.68390447	0.78525907
H	-0.34003663	1.70769024	-0.25691143
H	-2.95285082	8.12351418	-1.38563597

H	-4.02844858	7.74333811	-0.29723558
H	6.43798542	3.02707648	1.67282343
H	7.48427629	2.80990410	2.75703025
H	-1.13449919	6.69183302	0.59273773
H	-1.89485085	8.01750088	0.39425102
H	-6.47191811	3.95784092	0.54648668
H	-5.56398630	5.21869850	0.82394844
H	6.62655020	-0.76696944	0.95761079
H	6.65069056	0.72767395	1.30438519
H	-3.28137589	-4.25437307	0.02129978
H	-3.52649641	-4.90796423	-1.35584486
H	2.42197728	3.90714192	0.01784310
H	2.61059833	5.38313675	0.31000164
H	4.12879372	-6.87479496	2.01008749
H	4.22045946	-6.44914341	0.53477645
H	5.90410709	-5.61045599	1.89669037
H	6.32002211	-4.89063215	3.18185282
H	-1.33568060	4.43875170	0.79983497
H	-2.59782934	4.30902100	-0.12432983
H	7.71844769	-2.82704020	0.29135841
H	6.63185978	-3.30300736	1.28936577
H	-7.25024176	0.01475003	-2.26763487
H	-6.43684196	-0.89371401	-1.31796443
H	4.36835957	0.19520950	0.46500260
H	3.36207175	-0.29920006	-0.68076587
H	4.39670181	4.34121704	0.83622932
H	5.54493713	4.17176104	-0.11607368
H	0.60325819	-4.24067068	-1.02558994
H	0.51484644	-5.09878111	0.20088907
H	-0.88519168	-6.30798769	-0.71600688
H	-1.98181248	-7.12143183	0.09581801
H	-3.53562999	0.75825840	-1.94152868
H	-4.99999571	0.69511068	-1.55189085

H	-4.85096264	4.05104685	-0.78666884
H	-4.09577703	2.73114324	-1.10312927
H	-6.59958696	-3.43628407	-1.19093668
H	-5.11124611	-3.10598063	-1.13406944
H	0.25142449	8.25721931	-0.05498151
H	1.06541348	9.52504349	-0.56737399
H	1.62976038	-2.04289508	-1.77874577
H	0.14965077	-1.99983764	-1.40236247
H	-1.96568692	-1.49288082	-0.68019444
H	-2.23410058	-2.78129745	-1.44999337
H	2.91143322	1.75535083	-0.57530206
H	3.35465527	2.81390953	-1.58664763
H	3.35009956	-6.38927031	-1.67880702
H	2.22319508	-5.72246456	-0.87325496
H	-0.22432110	3.05255246	-2.20956302
H	1.07575452	2.30828810	-1.96224189
H	-3.29023027	-6.87959242	-3.34843159
H	-2.47472167	-6.92767715	-1.98631394
H	3.82850790	7.01500607	-1.24435353
H	2.41293645	7.40383148	-0.93694109
H	4.17266321	-4.09079456	-1.01084816
H	5.34631872	-3.17020798	-0.53253216
H	6.21470165	0.28036717	-1.10100484
H	5.19515657	0.02190623	-2.24648809
H	-1.04957855	4.93392706	-1.47198439
H	-1.63150692	4.53398037	-2.79157543
H	5.43402290	3.60459733	-2.74314952
H	5.54269457	2.28352451	-1.94229674
H	3.48879671	-1.07024360	-3.24115562
H	4.10231447	-2.01549649	-2.08746886
H	-1.26137626	0.90312672	-2.78093791
H	-1.81370819	-0.56430882	-2.68275738
H	-1.25447440	6.72318697	-2.56248784

H	-0.62720776	7.99040890	-2.13765931
H	-3.55488586	5.36778593	-3.56497884
H	-3.70963478	4.06565714	-2.81283021
O	1.68533599	-4.47887945	4.45765877
O	0.12504716	-2.55255127	3.25781393
O	-2.66092062	-2.08218479	2.94337344
O	-4.17119408	6.31102133	1.38805175
O	-2.50730157	-4.47153378	1.63047826
O	2.70782733	-7.04369831	3.30453777
O	0.78452277	-7.81115198	1.26232934
O	0.08654751	-5.24304962	1.80328763
O	-3.42934275	-7.02412510	1.19237101
O	-6.94718552	1.93703842	0.72096020
O	-0.17710359	3.86412954	1.76046276
O	-2.59988928	-0.44117629	0.67237157
O	1.10472655	-0.77061057	1.14915931
O	-0.52383679	1.32498145	0.60565621
O	-3.45660472	8.48932838	-0.60200447
O	7.14107466	2.39840126	1.96897256
O	-1.08523381	7.63791466	0.80860841
O	-6.12576723	4.78366232	0.19029558
O	6.12859249	0.04911248	0.81794959
O	-3.66595125	-4.08940935	-0.85945582
O	2.42192149	4.53223705	0.75691628
O	4.74671698	-6.83673573	1.24677336
O	6.40110207	-4.81043625	2.21972585
O	-1.92839611	4.97497940	0.20649855
O	6.83071518	-2.58469677	0.66429627
O	-6.67656851	0.02189106	-1.48364139
O	3.45862246	0.22060651	0.14006408
O	5.34338522	4.42731285	0.78658420
O	0.54888970	-5.17679119	-0.79649067
O	-1.30396926	-7.16229439	-0.56555033

O	-4.13234949	0.99194950	-1.23340738
O	-3.98037195	3.71825409	-1.03442824
O	-5.98423100	-2.72351980	-1.31775916
O	0.97663593	8.58580303	-0.66858292
O	0.99959642	-2.37627244	-1.14724827
O	-1.68042040	-1.99664998	-1.45568073
O	2.58068728	2.52576327	-1.05726910
O	3.16664314	-5.90276861	-0.88430637
O	0.11357777	2.17407608	-1.99934292
O	-3.18153930	-6.46636152	-2.47506976
O	2.96269989	6.64032316	-1.09530389
O	4.72276163	-3.35936570	-1.23183227
O	6.00119257	0.52471703	-2.03513193
O	-0.81928557	4.81648874	-2.38136125
O	5.19134760	3.17961907	-1.92798650
O	3.58408761	-1.19425762	-2.28085661
O	-2.06828070	0.33963242	-2.93640494
O	-1.48045361	7.64526701	-2.37613773
O	-3.23055339	4.47031593	-3.55944395

Table. S2. Optimized GFN2-xTB parameters

```
$Z= 8
  ao=2s2p
  lev    =   -22.531    -16.871
  exp    =       2.687        2.175
  GAM    =        0.407
  GAM3 =      -0.459
  KCNS =        0.113
  KCNP =      -0.133
  DPOL =      -4.068
  QPOL =      -0.245
  REPA =        2.193
  REPB =        6.567
  POLYS=    -12.005
  POLYP=      -2.691
  LPARP=        1.253
$end
$Z= 1
  ao=1s
  lev    =   -11.099
  exp    =       1.324
  GAM    =        0.452
  GAM3 =        0.754
  KCNS =      -0.570
  DPOL =        5.847
  QPOL =        0.029
  REPA =        1.880
  REPB =        0.881
  POLYS=      -0.892
$end
$pairpar
  1 1     1.003
```

1 8	0.999
1 17	0.873
8 8	0.949
8 17	1.200

$end
